# Choline chloride and amino acid solutions taste and hydration behavior with experimental thermodynamic properties and COSMO-PC-SAFT calculation

**DOI:** 10.1038/s41598-024-70275-z

**Published:** 2024-09-02

**Authors:** Mohammad Amin Morsali, Behrang Golmohammadi, Hemayat Shekaari

**Affiliations:** Department of Physical Chemistry, Faculty of Chemistry, University of Tabriz, 29 Bahman Boulevard, Tabriz, Iran

**Keywords:** Choline chloride, Amino acid, Thermodynamic properties, COSMO, PC-SAFT, Environmental chemistry, Green chemistry, Physical chemistry, Theoretical chemistry

## Abstract

Aqueous amino acid solutions have been introduced as dietary supplements for both animals and humans. This study investigates the physicochemical properties of the solutions containing amino acids (l-glycine, d,l-alanine, l-proline), choline chloride, and water at temperature range of 288.15 to 318.15 K. The results show that increasing concentrations of amino acids and choline chloride lead to higher solution densities. Analysis of apparent molar volume (*V*_*φ*_) and apparent molar isentropic compressibility (*κ*_*φ*_) reveals that *V*_*φ*_ values increase with choline chloride concentration and temperature, indicating enhanced solute–solvent interactions, while *κ*_*φ*_ values decrease, suggesting increased solution compression. Thermodynamic analysis using the Redlich-Mayer model and COSMO-based modeling provides insights into molecular interactions. However, COSMO-based parameters show high average relative deviation percentage (ARD %) values, indicating poor predictive performance for the density of these systems. In contrast, the ePC-SAFT equation of state effectively predicts the densities, particularly for l-proline-based solutions, which show very low ARD % values, indicating high accuracy. The ePC-SAFT model also performs reasonably well for l-glycine solutions but shows poorer results for d*,*l-alanine-based solutions. The study also examines the sweetness and saltiness criteria (ASV and ASIC) of these solutions. The ASV values, which serve as a sweetness criterion, are higher than the ideal range of 0.5 < ASV < 0.7, suggesting an overly sweet taste. The ASIC values follow a similar trend, indicating increased saltiness. To achieve an appropriate grade of sweetness and saltiness, dilution to lower concentrations of the solution is recommended. Furthermore, the use of choline chloride is found to increase salt intake and enhance the taste of salt, which can be beneficial in amino acid supplements used in animal food.

## Introduction

Pharmaceutical industry introduced the amino acid-based solutions as a new generation of the food supplements^1,2^. Research and development compartment of the pharmaceutical companies have been obligated to improve these supplements enrich them with inorganic ingredients such as calcium, potassium, and magnesium^3^. However, vitamins might be more effective besides the amino acids since they are compatible ingredient that improve the body functioning working together^4^. A lot of factors are needed to boost immunization system. Amino acids are one of these factors^5^. Nowadays some companies are trying to produce commercial syrup which contains useful and beneficial amino acids like glycine, proline and alanine^6^. These syrups provide necessary amino acids of human body and help it to work more efficiently^7^. Meanwhile choline chloride is another essential component in human daily diet^8^. Choline chloride has especial role in constituency of cell membrane^9^ and it’s the main component of pulmonary surfactants. Thus, producing syrup which contains choline chloride with amino acids could be the good way to boost immunization system and maximize the immunity^10^.

Studying about thermodynamic properties of choline chloride with amino acids lead researchers to find good composition of them in the best proportion^11^. Also, studying of these materials in molecular levels and deep understanding of their interactions would be helpful to find out the features of these components. Numerical statistical mechanic approach such as statistical associated fluid theory (SAFT)-base equation of states (EOS) are very well known and quite useful one for prediction of thermodynamic properties of the mixtures^12^. Perturbation chain-SAFT (PC-SAFT) is one of them based on Wertheim’s perturbation theory which used for chain molecules^13–15^. The hard chain contribution is the essential part of the PC-SAFT besides the dispersion contribution of the hard chains^16^. The ePC-SAFT is an extension of PC-SAFT for electrolyte mixtures with the Deby-Huckel contribution^17^, which accounts for the coulombic interactions. Association term is another contribution that should be considered for the aqueous electrolytes^18^. This equation of state could be used to predict different thermodynamic properties of the multi-components systems successfully^19^. Validation of this equation of state could be carried out using deviation analysis of predicted values with experimental data. The caloric values of a supplement are an important metric to evaluate it's energy for consumers^20^. Calorimetric experiment for a mixture is a time consuming and expensive procedure while it could be simply predicted using equation of states. The ePC-SAFT could provide caloric data such as enthalpy, entropy, and Gibbs free energy while it could be used for interpretation the existing interactions between the species in the mixture^21^.

There is other way to achieve the PC-SAFT data using microscopic approach. One of them is called COSMO model based on the σ-profile which is a crucial concept in COSMO-based thermodynamic, representing the charge distribution on a molecule's surface^22^. It acts as a unique fingerprint, indicating the likelihood of finding specific charge density values in fragmented segments. COSMO models, such as COSMO-RS and COSMO-SAC, utilize σ-profiles to predict thermodynamic properties and molecule-environment interactions. These profiles are obtained through computational methods, primarily employing density functional theory (DFT) calculations, which can be computationally expensive. To overcome this, alternative methods approximating σ-profiles are available in software tools and databases, enabling faster analysis, especially in high-throughput screening applications.

The thermodynamic properties (density and speed of sound) of ternary systems containing amino acid (l-glycine, d,l-alanine, and l-proline) + choline chloride in aqueous media have been investigated under atmospheric pressure at T = (288.15–318.15) K. The ePC-SAFT equation of state is used to achieve a suitable approach in microscopic scale from the studied systems. Also, the results of DFT and COSMO are used to interpret the microscopic approach for these systems. The results have been discussed with combination of classical and statistical thermodynamic^23^.

### Theoretical background

PC-SAFT (perturbed chain SAFT) uses chains of spheres to model fluids. This allows it to handle chain-like molecules and self-association (like hydrogen bonding) through second-order perturbation theory^24,25^. In a canonical ensemble (fixed temperature, volume, particles), the Helmholtz free energy relation provides a convenient way to derive thermodynamic properties for these complex fluids^26^:

1 $$a = a^{hc} + a^{disp} + a^{assoc}$$

2 $$a^{hc} = ma^{hs} - \sum\limits_{i}^{{}} {x_{i} (m_{i} - 1)\ln (g^{hs} (\sigma ))}$$

3 $$a^{disp} = - 2\pi I_{1} \overline{{m^{2} \varepsilon \sigma^{3} }} - \pi \rho mC_{1} I_{2} \overline{{m^{2} \varepsilon^{2} \sigma^{3} }}$$

4 $$a^{assoc} = \sum\limits_{i}^{{}} {x_{i} \left[ {\sum\limits_{{A_{i} }}^{{}} {(\ln X^{{A_{i} }} - \frac{{X^{{A_{i} }} }}{2})} + \frac{1}{2}M_{i} } \right]}$$

where, superscripts hc and disp, terms are used for the hard chain and dispersion which are given in the original version of PC-SAFT^13,27^. The symbol assoc was used for association contribution that uses the original SAFT equations^28,29^. The components of the equations have been described in the original paper as presented in these equations^13^. The density could be predicted using the following equation with iteration of pressure^30,31^,

5 $$Z = 1 + \rho \left( {\frac{{\partial \left( {a/K_{B} T} \right)}}{\partial \rho }} \right)$$

where, ρ and Z are the number density and compressibility factor, respectively. The main parameters of this equation of state are segment number, m, segment diameter, σ, dispersion energy, u_0_/K_B_, association energy, ε^AB^/K_B_, and effective association volume, κ^AB^. These parameters commonly obtained from the experimental vapor pressure or density data at different pressures and temperatures (P,T)^32^. However, there is some indirect methods to obtain these parameters^33^. Recently, a new methodology has been introduced that could be used to predict these parameters with density functional theory results^34^.

Electron density, a crucial molecular property, can be obtained using density functional theory^35^. However, another model, based on quantum mechanics and equilibrium thermodynamics, indirectly predicts various properties via chemical potentials^36^. This model has recently been applied to parameterize a popular equation of state for fluids. Both approaches share a common mathematical framework based on a specific system type (fixed temperature, volume, and particle number)^34^. This allows for a theoretical calculation of the equation of state parameters without needing real-world data. Researchers have exploited this link to derive segment diameters, a key parameter, from the other model's cavity and surface area outputs^37^.

6 $$\sigma = \frac{6V}{A}$$

7 $$m = \frac{A}{{\pi \sigma^{2} }}$$

8 $$\frac{{u_{0} }}{{K_{B} }} = \frac{a}{{\sigma^{6} }} + b$$

9 $$\frac{{\varepsilon^{AB} }}{{K_{B} }} = \frac{c}{{\sigma^{6} }} + d$$

where, V stands for the volume of cavity and A represents the corresponding area of cavity, and the a, b, c, and d symbols are the parameters that could be obtained from the correlation of the dispersion energy and association energy with the existing data in literature^34^. The other symbols stand for the PC-SAFT parameters^34^. It should be noted that the association volume has been considered between 0.01 and 0.03 for the studied systems.

The density and sound speed data of choline chloride in aqueous solutions of amino acids (l-glycine, l-proline, d,l-alanine) have been measured at different temperatures (288.15–318.15) K and ambient pressure. The thermodynamic properties were calculated from the experimental data and correlated with Redlich-Mayer model. Also, the densities of the studied mixtures have been predicted with PC-SAFT equation of state variations on the experimentally obtained parameters and COSMO-based obtained parameters. These results were used to determine the taste and hydration behavior and interpretation of the intermolecular interactions.

## Materials and methods

### Materials

All of the amino acids (l-glycine, d, l-alanine, l-proline have been purchased from Merck, and used without more purification while choline chloride was Sigma Aldrich. All of the materials purity were > 99%. Also, deionized ultrapure water with a specific conductance below 1 μS cm^−1^ was used to prepare the corresponding aqueous solutions of choline chloride in the presence of amino acids.

### Apparatus and procedure

An analytical balance (Shimadzu AW-220) with resolution of $\pm 1\times {10}^{-4}\text{g}$ and accuracy $\pm 2\times {10}^{-4}\text{g}$ was used to prepare the solution in molal-based concentration. A digital vibrating U-shaped densitometer (Anton Paar DSA5000) with resolution of ± 1 × 10^–6^ g cm^−3^ for density and 0.01 m s^−1^ for speed of sound while the uncertainty for these properties were 4 × 10^–5^ g cm^−3^ and 0.7 m s^−1^, respectively. The instrument has been calibrated with air and distilled water while the frequency for speed of sound measurement was 3 MHz.

### Theoretical calculations

A specific computational method (mentioning the general class, GGA, is optional) within a popular software package (Dmol3) was used to optimize molecular geometry. This method has been suggested by the software developers as potentially effective for studying real solvents. The optimization relied on a particular set of calculations (DFT) that provided the necessary data (COSMO results).

## Results and discussion

### Thermodynamic properties

The density (*d*) and speed of sound (*u*) data of the aqueous solutions of the amino acids (l-glycine, d,l-alanine, l-proline) and choline chloride were measured under atmospheric pressure (*P *= 0.086 MPa) at temperature range *T* = (288.15–318.15) K. These data are given in Table 1. The experimental density data of aqueous amino acid solutions have been compared with literature data as shown in Figs. S1–S3 (supporting information). The data was in good agreement with those reported in the literature data.

**Table 1:** The density (*d*), speed of sound (*u*), and isentropic compressibility (*κ*_*S*_) data of the ternary solutions (Water + cqholine chloride + amino acid (l-glycine, d,l-alanine, l-proline)) under pressure (*P* = 871.5 hPa) at temperature range (288.15–318.15) K.

*m*_*ChCl*_	*d*				*u*				10^10^*κ*_*S*_			
mol kg^−1^	kg m^−3^				m s^−1^				Pa^−1^			
	288.15	298.15	308.15	318.15	288.15	298.15	308.15	318.15	288.15	298.15	308.15	318.15
	K				K				K			
*m*_*L-Glycine*_ = 0.0500 mol kg^−1^												
0.0000	1000.74	998.63	995.52	991.45	1469.46	1499.29	1521.94	1538.04	4.63	4.45	4.34	4.26
0.0499	1001.59	999.45	996.31	992.22	1474.84	1504.27	1526.55	1542.33	4.59	4.42	4.31	4.24
0.0998	1002.41	1000.23	997.08	992.98	1480.02	1509.02	1531.11	1546.50	4.55	4.39	4.28	4.21
0.1494	1003.22	1001.00	997.84	993.71	1484.99	1513.74	1535.18	1550.47	4.52	4.36	4.25	4.19
0.1986	1003.97	1001.74	998.57	994.42	1489.99	1518.37	1539.50	1554.59	4.49	4.33	4.23	4.16
0.2490	1004.75	1002.49	999.26	995.10	1495.05	1522.95	1543.77	1558.49	4.45	4.30	4.20	4.14
0.2950	1005.46	1003.18	999.91	995.75	1499.68	1527.18	1547.69	1562.13	4.42	4.27	4.18	4.12
*m*_*L-Glycine*_ = 0.1000 mol kg^−1^												
0.0000	1002.35	1000.21	997.14	993.29	1472.19	1501.90	1524.35	1540.36	4.60	4.43	4.32	4.24
0.0498	1003.16	1000.99	997.90	994.02	1477.57	1506.85	1528.90	1544.63	4.57	4.40	4.29	4.22
0.0988	1003.95	1001.75	998.64	994.73	1482.63	1511.56	1533.26	1548.75	4.53	4.37	4.26	4.19
0.1495	1004.75	1002.51	999.39	995.46	1487.78	1516.33	1537.61	1552.86	4.50	4.34	4.23	4.17
0.1985	1005.53	1003.27	1000.13	996.16	1492.79	1520.91	1541.90	1556.82	4.46	4.31	4.21	4.14
0.2498	1006.34	1004.01	1000.86	996.91	1497.91	1525.63	1546.25	1560.86	4.43	4.28	4.18	4.12
0.2987	1007.08	1004.74	1001.55	997.55	1502.85	1530.12	1550.43	1564.75	4.40	4.25	4.15	4.09
*m*_*L-Glycine*_ = 0.1500 mol kg^−1^												
0.0000	1003.98	1001.79	998.70	994.81	1474.92	1504.38	1526.70	1542.63	4.58	4.41	4.30	4.22
0.0498	1004.77	1002.56	999.43	995.48	1480.26	1509.37	1531.37	1547.03	4.54	4.38	4.27	4.20
0.0999	1005.57	1003.31	1000.15	996.15	1485.30	1514.20	1535.92	1551.17	4.51	4.35	4.24	4.17
0.1499	1006.35	1004.07	1000.86	996.79	1490.52	1518.90	1540.30	1555.54	4.47	4.32	4.21	4.15
0.2000	1007.14	1004.83	1001.59	997.44	1495.65	1523.60	1544.61	1559.82	4.44	4.29	4.18	4.12
0.2483	1007.88	1005.55	1002.27	998.05	1500.53	1528.08	1548.61	1563.45	4.41	4.26	4.16	4.10
0.2993	1008.64	1006.27	1002.92	998.64	1505.53	1532.60	1552.99	1567.56	4.37	4.23	4.13	4.08
*m*_*DL-Alanine*_ = 0.0500 mol kg^−1^												
0.0000	1000.54	998.46	995.48	991.59	1470.04	1499.79	1522.35	1538.46	4.62	4.45	4.33	4.26
0.0497	1001.37	999.25	996.25	992.31	1475.25	1504.70	1526.87	1542.72	4.59	4.42	4.31	4.23
0.0994	1002.19	1000.01	996.96	993.03	1480.47	1509.30	1531.20	1546.75	4.55	4.39	4.28	4.21
0.1498	1002.99	1000.80	997.71	993.72	1485.73	1514.14	1535.69	1551.01	4.52	4.36	4.25	4.18
0.1998	1003.77	1001.54	998.43	994.43	1490.47	1518.74	1539.73	1554.83	4.48	4.33	4.22	4.16
0.2496	1004.52	1002.26	999.13	995.10	1495.29	1523.11	1543.91	1559.11	4.45	4.30	4.20	4.13
0.2996	1005.30	1003.03	999.87	995.78	1500.45	1527.91	1548.41	1563.05	4.42	4.27	4.17	4.11
*m*_*DL-Alanine*_ = 0.0999 mol kg^−1^												
0.0000	1001.99	999.88	996.83	992.98	1473.63	1503.12	1525.44	1541.72	4.60	4.43	4.44	4.24
0.0499	1002.84	1000.68	997.60	993.74	1478.86	1508.09	1530.02	1545.99	4.56	4.39	4.28	4.21
0.0989	1003.65	1001.46	998.35	994.47	1484.02	1512.77	1534.36	1549.89	4.52	4.36	4.25	4.19
0.1500	1004.46	1002.24	999.12	995.21	1489.30	1517.60	1538.83	1554.03	4.49	4.33	4.23	4.16
0.2003	1005.25	1002.99	999.85	995.92	1494.39	1522.38	1543.17	1558.11	4.45	4.30	4.20	4.14
0.2503	1006.02	1003.72	1000.57	996.62	1499.53	1527.00	1547.47	1561.83	4.42	4.27	4.17	4.11
0.2993	1006.77	1004.43	1001.27	997.27	1504.38	1531.43	1551.58	1565.41	4.39	4.25	4.15	4.09
*m*_*DL-Alanine*_ = 0.1499 mol kg^−1^												
0.0000	1003.42	1001.28	998.21	994.35	1477.13	1506.35	1528.43	1544.15	4.57	4.40	4.29	4.22
0.0506	1004.22	1002.05	998.95	995.08	1482.41	1511.35	1533.05	1548.49	4.53	4.37	4.26	4.19
0.1007	1005.02	1002.80	999.70	995.79	1487.63	1516.05	1537.36	1552.52	4.50	4.34	4.23	4.17
0.1520	1005.83	1003.57	1000.46	996.52	1492.90	1520.94	1541.94	1556.67	4.46	4.31	4.20	4.14
0.2023	1006.63	1004.33	1001.20	997.26	1498.04	1525.60	1546.21	1560.71	4.43	4.28	4.18	4.12
0.2513	1007.40	1005.07	1001.90	997.98	1502.89	1530.11	1550.48	1564.51	4.39	4.25	4.15	4.09
0.3030	1008.21	1005.85	1002.66	998.71	1508.20	1534.87	1554.78	1568.11	4.36	4.22	4.13	4.07
*m*_*L-Proline*_ = 0.0501 mol kg^−1^												
0.0000	1000.69	998.59	995.65	991.88	1470.83	1500.36	1522.76	1540.53	4.62	4.45	4.33	4.25
0.0481	1001.51	999.37	996.40	992.60	1475.83	1505.07	1527.18	1544.31	4.58	4.42	4.30	4.22
0.0981	1002.31	1000.15	997.12	993.31	1480.95	1509.82	1531.51	1547.91	4.55	4.39	4.28	4.20
0.1476	1003.11	1000.92	997.84	993.95	1486.10	1514.52	1536.22	1551.90	4.51	4.36	4.25	4.18
0.1950	1003.88	1001.65	998.54	994.62	1490.90	1519.02	1540.15	1555.23	4.48	4.33	4.22	4.16
0.2442	1004.67	1002.40	999.26	995.41	1495.95	1523.64	1544.23	1558.70	4.45	4.30	4.20	4.13
0.2912	1005.39	1003.08	999.94	996.00	1500.63	1528.33	1548.52	1562.72	4.42	4.27	4.17	4.11
*m*_*L-Proline*_ = 0.0962 mol kg^−1^												
0.0000	1002.41	1000.27	997.20	993.34	1474.90	1504.3	1526.0	1541.7	4.59	4.42	4.31	4.24
0.0498	1003.24	1001.07	997.98	994.09	1480.26	1509.3	1530.6	1546.0	4.55	4.39	4.28	4.21
0.0997	1004.08	1001.83	998.74	994.80	1485.41	1513.7	1534.9	1549.9	4.51	4.36	4.25	4.18
0.1502	1004.86	1002.60	999.47	995.53	1490.52	1518.5	1539.4	1554.2	4.48	4.33	4.22	4.16
0.1992	1005.64	1003.35	1000.18	996.19	1495.42	1523.1	1543.7	1559.1	4.45	4.30	4.20	4.13
0.2495	1006.40	1004.12	1000.88	996.90	1500.43	1527.7	1547.9	1562.7	4.41	4.27	4.17	4.11
0.3112	1007.18	1004.84	1001.65	997.67	1505.47	1532.24	1552.19	1566.16	4.38	4.24	4.14	4.09
*m*_*L-Proline*_ = 0.1488 mol kg^−1^												
0.0000	1004.01	1001.84	998.74	994.86	1479.41	1508.21	1529.88	1545.26	4.55	4.38	4.28	4.21
0.0486	1004.81	1002.61	999.48	995.59	1484.39	1512.86	1534.18	1549.29	4.52	4.36	4.37	4.19
0.0991	1005.63	1003.38	1000.24	996.34	1489.52	1517.41	1538.33	1553.18	4.48	4.33	4.34	4.16
0.1477	1006.40	1004.11	1000.96	997.06	1494.43	1522.04	1542.61	1557.30	4.45	4.30	4.31	4.14
0.1997	1007.23	1004.90	1001.73	997.82	1499.79	1526.93	1547.18	1561.38	4.41	4.27	4.28	4.11
0.2492	1007.98	1005.62	1002.43	998.50	1504.72	1531.42	1551.35	1565.50	4.38	4.24	4.25	4.09
0.2955	1008.70	1006.32	1003.10	999.18	1509.40	1535.72	1555.31	1568.98	4.35	4.21	4.23	4.07

The standard uncertainties for molality, temperature and pressure were* u* (*m*) = 0.002 mol kg^−1^, *u* (*T*) = 0.02 K, *u* (*P*) = 10 hPa, respectively with level of confidence 0.68. The combined standard uncertainty for density and speed of sound were, *u*_*c*_ (*d*) = 0.04 kg m^−3^, *u*_*c*_ (*u*) = 0.6 m s^−1^ with level of confidence 0.95.

**Figure 1:**
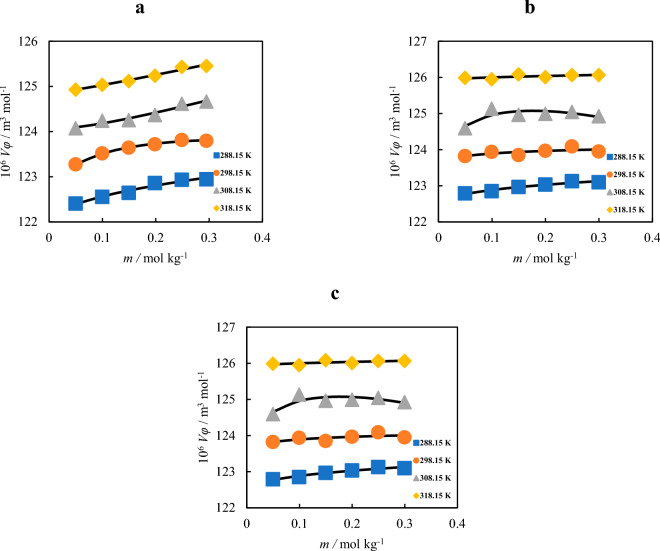
The apparent molar volume of choline chloride versus its molality in the presence of 0.05 mol kg^−1^: (**a**) l-glycine, (**b**) l-alanine, and (**c**) l-proline at different temperatures.

Also, the density of the solutions has been increased with addition of the amino acid and choline chloride content and decreased with increasing temperature. The apparent molar volumes and apparent molar isentropic compressibility values of the choline chloride in the amino acid + water solutions were evaluated using the following equation^38^,

10 $$V_{\varphi } = \frac{M}{d} - \left[ {\frac{{\left( {d - d_{0} } \right)}}{{mdd_{0} }}} \right]$$

11 $$\kappa_{s} = \frac{1}{{du^{2} }}$$

12 $$\kappa_{\varphi } = \frac{{\kappa_{S} d_{0} - d\kappa_{{S_{0} }} }}{{mdd_{0} }} + \frac{{\kappa_{S} M}}{d}$$

where, the symbols *m*, *M*, *d*, *d*_*0*_, *u*, *κ*_*s*_, *κ*_*s0*_ are the molality, molar mass of choline chloride, density of the solution, density of solvent, speed of sound, isentropic compressibility of the solution, and isentropic compressibility of the solvent. As could be seen in Table 1 by increasing of the concentration of amino acids, the *κ*_*s*_ is decreased. Also, the *V*_*φ*_ values are plotted in Fig. 1 at different temperatures.

It’s clear that when the concentrationof the increased the compression will be harder than before. Also, based on Table 1 the isentropic compressibility is decreased while the temperature is going up. It’s obvious by increasing of temperature the density is decreased but the speed of sound is increased too. Based on equation. the *κ*_*s*_ and sound velocity has inversion relationship so in total *κ*_*s*_ is decreased. The thermodynamic properties of the studied solutions have been studied to achieve a good aspect of view around them. Accordingly, the apparent molar volume and apparent molar isentropic compressibility of the choline chloride have been given with *V*_*φ*_ and *κ*_*φ*_. These values are given in Table 2.

**Table 2:** The apparent molar volume and apparent molar isentropic compressibility of the choline chloride in the ternary systems containing (water + choline chloride + amino acid (l-glycine, d,l-alanine, l-proline) under *P* = 865 hPa at *T* = (288.15–318.15) K.

*m*_*ChCl*_/mol kg^−1^	10^6^ *V*_*φ*_/m^3^ mol^−1^				10^14^ *κ*_φ_/m^3^ mol^−1^ Pa^−1^			
	288.15 K	298.15 K	308.15 K	318.15 K	288.15 K	298.15 K	308.15 K	318.15 K
*m*_*L-Glycine*_ = 0.0500 mol kg^−1^								
0.0499	122.41	123.27	124.08	124.93	− 1.91	− 1.18	− 0.61	− 0.16
0.0998	122.55	123.52	124.24	125.04	− 1.77	− 1.03	− 0.57	− 0.09
0.1494	122.64	123.64	124.25	125.12	− 1.64	− 0.97	− 0.39	0.00
0.1986	122.86	123.72	124.37	125.24	− 1.59	− 0.93	− 0.37	0.00
0.2490	122.93	123.81	124.62	125.43	− 1.54	− 0.86	− 0.31	0.06
0.2950	122.94	123.80	124.67	125.45	− 1.52	− 0.84	− 0.29	0.09
*m*_*L-Glycine*_ = 0.1000 mol kg^−1^								
0.0498	123.09	123.81	124.61	125.58	− 1.82	− 1.08	− 0.48	− 0.07
0.0988	123.02	123.86	124.65	125.60	− 1.67	− 0.98	− 0.41	− 0.02
0.1495	123.02	123.93	124.65	125.59	− 1.59	− 0.90	− 0.33	0.03
0.1985	122.97	123.83	124.54	125.54	− 1.56	− 0.87	− 0.33	0.06
0.2498	122.90	123.93	124.60	125.40	− 1.52	− 0.82	− 0.29	0.09
0.2987	122.96	123.89	124.65	125.56	− 1.49	− 0.79	− 0.27	0.12
*m*_*L-Glycine*_ = 0.1500 mol kg^−1^								
0.0498	123.19	123.96	125.01	126.66	− 1.71	− 1.07	− 0.55	− 0.09
0.0999	123.05	124.01	125.07	126.65	− 1.52	− 0.96	− 0.46	0.05
0.1499	123.07	123.93	125.07	126.72	− 1.53	− 0.89	− 0.38	0.01
0.2000	122.98	123.85	124.96	126.73	− 1.51	− 0.86	− 0.33	0.02
0.2483	123	123.83	124.95	126.73	− 1.48	− 0.82	− 0.26	0.12
0.2993	123.04	123.9	125.12	126.91	− 1.42	− 0.75	− 0.23	0.16
*m*_*DL-Alanine*_ = 0.0500 mol kg^−1^								
0.0497	122.79	123.82	124.59	125.99	− 1.68	− 1.07	− 0.48	− 0.05
0.0994	122.85	123.94	125.13	125.95	− 1.68	− 0.88	− 0.33	0.06
0.1498	122.97	123.85	124.96	126.08	− 1.66	− 0.90	− 0.35	0.04
0.1998	123.03	123.97	124.99	126.01	− 1.50	− 0.83	− 0.24	0.13
0.2496	123.13	124.09	125.04	126.07	− 1.43	− 0.75	− 0.20	0.09
0.2996	123.10	123.95	124.93	126.06	− 1.45	− 0.78	− 0.25	0.12
*m*_*DL-Alanine*_ = 0.0999 mol kg^−1^								
0.0499	122.42	123.41	124.33	125.12	− 1.67	− 1.12	− 0.52	− 0.09
0.0989	122.49	123.47	124.36	125.20	− 1.68	− 1.00	− 0.44	0.06
0.1500	122.66	123.63	124.41	125.25	− 1.63	− 0.93	− 0.38	0.09
0.2003	122.76	123.73	124.50	125.34	− 1.57	− 0.90	− 0.34	0.11
0.2503	122.83	123.83	124.54	125.43	− 1.55	− 0.85	− 0.31	0.19
0.2993	122.88	123.87	124.56	125.54	− 1.50	− 0.80	− 0.28	0.26
*m*_*DL-Alanine*_ = 0.1499 mol kg^−1^								
0.0506	123.23	124.19	125.00	125.83	− 1.53	− 0.97	− 0.41	− 0.02
0.1007	123.16	124.14	124.81	125.79	− 1.53	− 0.83	− 0.28	0.11
0.1520	123.05	124.10	124.73	125.68	− 1.51	− 0.82	− 0.31	0.13
0.2023	122.96	124.01	124.66	125.49	− 1.549	− 0.78	− 0.26	0.14
0.2513	122.92	123.93	124.66	125.36	− 1.44	− 0.76	− 0.26	0.17
0.3030	122.86	123.81	124.58	125.30	− 1.45	− 0.75	− 0.23	0.26
*m*_*L-Proline*_ = 0.0501 mol kg^−1^								
0.0481	122.39	123.45	124.39	125.36	− 1.66	− 1.05	− 0.55	0.49
0.0981	122.74	123.63	124.94	125.75	− 1.58	− 0.95	− 0.35	0.50
0.1476	122.80	123.67	125.03	126.19	− 1.59	− 0.92	− 0.46	0.43
0.1950	122.79	123.72	124.96	126.08	− 1.56	− 0.90	− 0.36	0.51
0.2442	122.76	123.70	124.87	125.62	− 1.56	− 0.89	− 0.30	0.52
0.2912	122.83	123.80	124.85	125.84	− 1.52	− 0.92	− 0.34	0.43
*m*_*L-Proline*_ = 0.0962 mol kg^−1^								
0.0498	122.59	123.40	124.18	125.17	− 1.81	− 1.09	− 0.60	− 0.10
0.0997	122.36	123.77	124.28	125.52	− 1.70	− 0.85	− 0.35	0.09
0.1502	122.72	123.79	124.52	125.49	− 1.58	− 0.77	− 0.34	0.05
0.1992	122.74	123.76	124.57	125.70	− 1.52	− 0.78	− 0.34	0.19
0.2495	122.86	123.70	124.69	125.64	− 1.46	− 0.75	− 0.28	0.10
0.3112	123.45	124.33	125.07	125.91	− 1.16	− 0.48	− 0.05	0.31
*m*_*L-Proline*_ = 0.1488 mol kg^−1^								
0.0486	122.67	123.56	124.43	125.09	− 1.44	− 0.83	− 0.29	0.07
0.0991	122.69	123.70	124.39	125.04	− 1.41	− 0.65	− 0.12	0.22
0.1477	122.76	123.76	124.45	125.01	− 1.38	− 0.70	− 0.17	0.13
0.1997	122.71	123.72	124.42	125.02	− 1.41	− 0.71	− 0.20	0.17
0.2492	122.81	123.77	124.48	125.13	− 1.37	− 0.68	− 0.18	0.14
0.2955	122.77	123.72	124.45	125.04	− 1.37	− 0.68	− 0.18	0.18

The standard uncertainties for molality, temperature and pressure were* u* (*m*) = 0.002 mol kg^−1^, *u* (*T*) = 0.02 K, *u* (*P*) = 10 hPa, respectively with level of confidence 0.68. The combined standard uncertainty for apparent molar volume and apparent molar isentropic compressibility were, 10^6^ *u*_*c*_ (*V*^*0*^_*φ*_) = 0.4 m^3^ mol^−1^, 10^14^ *u*_*c*_ (*κ*^*0*^_*φ*_) = 0.7 m^3^ mol^−1^ Pa^−1^with level of confidence 0.95.

The apparent molar properties have been correlated using Redlich-Mayer model as given by following equations:

13 $$V_{\varphi } = V_{\varphi }^{0} + S_{v} m^{1/2} + B_{v} m$$

14 $$\kappa_{\varphi } = \kappa_{\varphi }^{0} + S_{k} m^{1/2} + B_{k} m$$

where, *V*_*φ*_^*0*^, *S*_*v*_, and *B*_*v*_ values are given in Table 3, for the binary solutions. The *V*_*φ*_^*0*^ values are a criterion of solute–solvent interaction, while the *S*_*v*_ value indicating the solute–solute interactions, and *B*_*v*_ is an adjustable parameter. The *V*_*φ*_^*0*^ values of the studied solutions are increased by concentration of choline chloride and at higher temperature in the binary solutions. the *κ*_*φ*_^*0*^ is the partial molar isentropic compressibility and *S*_*κ*_ and *B*_*κ*_ are the empirical parameters of the equation. The obtained parameters for the investigated solutions are listed in Table 3 for the studied solutions.

**Table 3:** Temperature dependency of *V*^*0*^_*φ*_, *S*_*v*_, *B*_*v*_,* κ*^*0*^_*φ*_, *S*_*κ*_, *B*_*κ*_, *E*^*0*^_*φ*_, *α*, with corresponding standard deviation of Redlich-Mayer for apparent molar volume (*σV*^*0*^_*φ*_) and apparent molar isentropic compressibility (*σκ*^*0*^_*φ*_), and the Hepler constant {*(∂*^*2*^* V*^*0*^_*φ*_*/∂T*^*2*^*)*_*P*_}.

*T*	10^6^ *V*^*0*^_*φ*_	10^6^ Sv	10^6^ *B*_*v*_	10^6^ *σ V*^*0*^_*φ*_	10^6^ *E*^*0*^_*φ*_	10^4^ *α*	10^6^ *(∂*^*2*^* V*^*0*^_*φ*_*/∂T*^*2*^*)*_*P*_	10^14^ *κ*^*0*^_*φ*_	10^14^ *S*_*κ*_	10^14^ *B*_*κ*_	10^14^ *σκ*^*0*^_*φ*_
K	m^3^ mol^−1^	m^3^ mol^−1^ kg^−1/2^	m^3^ mol^−1^ kg^−1^	m^3^ mol^−1^	m^3^ mol^−1^ K^−1^	K^−1^	m^3^ mol^−1^ K^−2^	m^3^ mol^−1^ Pa^−1^	m^3^ kg^1/2^ mol^−3/2^ Pa^−1^	m^3^ kg mol^−2^ Pa^−1^	m^3^ mol^−1^ Pa^−1^
*m*_*L-Glycine*_ = 0.0500 mol kg^−1^											
288.15 K	121.97	1.92	− 0.11	0.04	0.09	7.04	0.0013	− 2.47	− 2.47	− 2.28	0.01
298.15 K	122.42	4.70	− 3.94	0.02	0.10	8.05		− 1.59	− 1.59	− 1.49	0.01
308.15 K	124.17	− 1.27	4.07	0.03	0.11	8.96		− 0.96	− 0.96	− 0.70	0.04
318.15 K	124.88	− 0.42	2.82	0.05	0.12	9.92		− 0.37	− 0.37	− 0.23	0.02
*m*_*L-Glycine*_ = 0.1000 mol kg^−1^											
288.15 K	123.33	− 1.30	− 1.30	0.03	0.03	2.43	0.0034	− 2.31	− 2.31	− 2.24	0.01
298.15 K	123.59	1.31	1.31	0.04	0.06	5.14		− 1.41	− 1.41	− 1.08	0.01
308.15 K	124.71	− 0.51	− 0.51	0.06	0.10	7.79		− 0.75	− 0.75	− 0.95	0.01
318.15 K	125.64	− 0.09	− 0.09	0.04	0.13	10.40		− 0.23	− 0.23	− 0.11	0.01
*m*_*L-Glycine*_ = 0.1500 mol kg^−1^											
288.15 K	123.68	− 2.91	3.16	0.03	0.01	0.60	0.0068	− 2.08	2.16	2.16	0.04
298.15 K	124.11	− 0.60	0.25	0.05	0.08	6.05		− 1.34	1.34	1.34	0.01
308.15 K	125.17	− 0.81	1.11	0.04	0.14	11.41		− 0.77	0.96	0.96	0.01
318.15 K	126.96	− 2.09	3.53	0.07	0.21	16.59		− 0.17	0.36	0.36	0.04
*m*_*DL-Alanine*_ = 0.0500 mol kg^−1^											
288.15 K	122.48	1.41	− 0.41	0.03	− 0.02	− 0.02	0.0081	− 1.625	− 0.851	2.279	0.03
298.15 K	123.61	1.15	− 0.79	0.07	0.06	0.06		− 1.522	2.561	− 2.194	0.01
308.15 K	123.17	9.04	− 10.73	0.04	0.14	0.14		− 0.923	2.49	− 2.252	0.04
318.15 K	125.92	0.25	0.06	0.10	0.22	0.22		− 0.396	1.992	− 1.946	0.02
*m*_*DL-Alanine*_ = 0.0999 mol kg^−1^											
288.15 K	122.12	1.21	0.41	0.03	0.11	9.27	− 0.0007	− 1.595	− 0.761	− 0.761	0.01
298.15 K	123.17	0.78	0.97	0.03	0.11	8.60		− 1.432	1.609	1.609	0.01
308.15 K	124.25	0.10	0.89	0.01	0.10	7.94		− 0.759	1.186	1.186	0.00
318.15 K	125.16	− 0.77	2.64	0.02	0.09	7.30		− 0.334	1.231	1.231	0.03
*m*_*DL-Alanine*_ = 0.1499 mol kg^−1^											
288.15 K	123.45	123.45	123.45	0.02	0.11	9.07	− 0.0017	− 1.49	− 0.39	0.87	0.01
298.15 K	124.00	124.00	124.00	0.01	0.10	7.65		− 1.338	2.074	− 1.85	0.03
308.15 K	125.58	125.58	125.58	0.04	0.08	6.18		− 0.662	1.441	− 1.244	0.02
318.15 K	125.79	125.79	125.79	0.02	0.06	4.80		− 0.225	1.064	− 0.419	0.03
*m*_*L-Pronine*_ = 0.0501 mol kg^−1^											
288.15 K	121.29	6.77	− 7.44	0.05	0.13	10.91	− 0.0062	− 1.812	− 1.812	− 0.568	0.03
298.15 K	123.00	2.55	− 2.11	0.03	0.07	5.74		− 1.478	− 1.478	− 2.734	0.02
308.15 K	122.35	12.91	− 15.53	0.17	0.01	0.73		− 0.873	− 0.873	− 1.678	0.05
318.15 K	122.83	15.67	− 19.15	0.06	− 0.05	− 4.30		− 0.365	− 0.365	− 4.826	0.04
*m*_*L-Proline*_ = 0.0962 mol kg^−1^											
288.15 K	123.89	− 9.20	14.87	0.11	− 0.02	− 1.42	0.0033	− 1.69	− 1.345	3.92	0.05
298.15 K	123.57	− 1.22	4.04	0.18	0.02	1.22		− 1.332	1.128	0.47	0.08
308.15 K	124.34	− 1.94	5.65	0.08	0.05	3.83		− 0.764	0.744	0.741	0.07
318.15 K	124.68	2.62	− 0.93	0.06	0.08	6.44		− 0.293	0.955	0.077	0.07
*m*_*L-Proline*_ = 0.1488 mol kg^−1^											
288.15 K	122.57	0.48	− 0.14	0.03	0.07	5.34	0.0021	− 1.529	0.463	− 0.345	0.01
298.15 K	122.99	3.48	− 3.91	0.02	0.09	7.05		− 1.22	2.462	− 2.78	0.04
308.15 K	124.47	− 0.34	0.60	0.04	0.11	8.66		− 0.671	2.463	− 2.958	0.04
318.15 K	125.31	− 1.47	1.90	0.03	0.13	10.29		− 0.159	1.568	− 1.81	0.04

The *κ*_*φ*_^*0*^ values increase with the concentration of amino acids. Also, the *V*_*φ*_^*0*^ values temperature dependency are fitted with a second-degree polynomial equation,

15 $$V_{\varphi }^{0} = A + BT + CT^{2}$$

the empirical parameters of *A*, *B*, and *C* have been used to calculate the standard apparent molar expansibility at constant pressure *E*_*φ*_^*0*^ using following equation^38^

16 $$E_{\varphi }^{0} = \left( {\frac{{\partial V_{\varphi }^{0} }}{\partial T}} \right)_{p} = B + 2CT$$

The values of *E*_*φ*_^*0*^are given in Table 3. The apparent isobaric thermal expansion was evaluated by the following equation^38^,

17 $$\alpha = \frac{{E_{\varphi }^{0} }}{{V_{\varphi }^{0} }}$$

The calculated values of *α* for choline chloride are given in Table 3. The value of *α* is a criterion for the apparent molar volume temperature dependency and its response to increment of temperature. The large value of this factor means the system volume is sensitive to temperature change. The pressure would also break the solvent structure and the same reason suggests that the heat capacity decrease. Hepler’s^39^ determined relation for structure making or breaking behavior of a solute in a solution is given by the following equations,

18 $$\left( {\frac{{\partial C_{P} }}{\partial P}} \right)_{T} = - T\left( {\frac{{\partial^{2} V_{\varphi }^{0} }}{{\partial T^{2} }}} \right)_{P} = - 2CT$$

where, $\left({\partial }^{2}{V}_{\varphi }^{0}/{\partial T}^{2}\right)$ is the constant for the choline chloride are given in Table 3. Negative values of this parameter mean the choline chloride have structure breaker behavior while otherwise it would be structure maker. The taste behavior of the solutions has been investigated using the apparent specific volume (*ASV*) and apparent specific isentropic compressibility (*ASIC*) by following relation^40^:

19 $$ASV = \frac{{V_{\varphi } }}{M}$$

20 $$ASIC = \frac{{\kappa_{\varphi } }}{M}$$

The symbols of these equations have been stated earlier. The results for the *ASV* and *ASIC* have been given in Table 4.

**Table 4:** The *ASV* values variation with molality of the aqueous solution of choline chloride in the presence of the amino acids at different temperatures (288.15–318. 15) K.

*m*/mol kg^−1^	*ASV*/m^3^ kg^−1^				*ASIC/*m^3^ kg^−1^ Pa^−1^			
	288.15 K	298.15 K	308.15 K	318.15 K	288.15 K	298.15 K	308.15 K	318.15 K
*m*_*L-Glycine*_ = 0.0500 mol kg^−1^								
0.0499	0.877	0.883	0.889	0.895	− 0.0140	− 0.0085	− 0.0044	− 0.0012
0.0998	0.878	0.885	0.89	0.896	− 0.0130	− 0.0074	− 0.0041	− 0.0007
0.1494	0.878	0.886	0.89	0.896	− 0.0120	− 0.0070	− 0.0028	0.0000
0.1986	0.880	0.886	0.891	0.897	− 0.0110	− 0.0067	− 0.0027	− 0.0001
0.2490	0.880	0.887	0.893	0.898	− 0.0110	− 0.0062	− 0.0023	0.0005
0.2950	0.881	0.887	0.893	0.899	− 0.0110	− 0.0060	− 0.0021	0.0006
*m*_*L-Glycine*_ = 0.1000 mol kg^−1^								
0.0498	0.882	0.887	0.892	0.899	− 0.0130	− 0.0078	− 0.0035	− 0.0005
0.0988	0.881	0.887	0.893	0.900	− 0.0120	− 0.0070	− 0.0030	− 0.0002
0.1495	0.881	0.888	0.893	0.899	− 0.0110	− 0.0065	− 0.0024	0.0003
0.1985	0.881	0.887	0.892	0.899	− 0.0110	− 0.0062	− 0.0024	0.0005
0.2498	0.880	0.888	0.892	0.898	− 0.0110	− 0.0059	− 0.0021	0.0007
0.2987	0.881	0.887	0.893	0.899	− 0.0110	− 0.0057	− 0.0019	0.0009
*m*_*L-Glycine*_ = 0.1500 mol kg^−1^								
0.0498	0.881	0.888	0.895	0.907	− 0.0120	− 0.0077	− 0.0039	− 0.0007
0.0999	0.881	0.888	0.896	0.907	− 0.0110	− 0.0069	− 0.0034	0.0004
0.1499	0.881	0.888	0.896	0.908	− 0.0110	− 0.0064	− 0.0028	0.0001
0.2000	0.88	0.887	0.895	0.908	− 0.0110	− 0.0062	− 0.0024	0.0001
0.2483	0.881	0.887	0.895	0.908	− 0.0110	− 0.0059	− 0.0019	0.0009
0.2993	0.881	0.887	0.896	0.909	− 0.0100	− 0.0054	− 0.0017	0.0012
*m*_*DL-Alanine*_ = 0.0500 mol kg^−1^								
0.0497	0.879	0.887	0.892	0.902	− 0.0121	− 0.0077	− 0.0035	− 0.0004
0.0994	0.88	0.888	0.896	0.902	− 0.0121	− 0.0063	− 0.0024	0.0005
0.1498	0.881	0.887	0.895	0.903	− 0.0119	− 0.0065	− 0.0025	0.0003
0.1998	0.881	0.888	0.895	0.902	− 0.0108	− 0.0060	− 0.0017	0.0010
0.2496	0.882	0.889	0.896	0.903	− 0.0102	− 0.0054	− 0.0015	0.0006
0.2996	0.882	0.888	0.895	0.903	− 0.0104	− 0.0057	− 0.0018	0.0009
*m*_*DL-Alanine*_ = 0.0999 mol kg^−1^								
0.0499	0.877	0.884	0.890	0.896	− 0.0120	− 0.0080	− 0.0038	− 0.0007
0.0989	0.877	0.884	0.891	0.897	− 0.0120	− 0.0072	− 0.0032	0.0005
0.1500	0.878	0.885	0.891	0.897	− 0.0120	− 0.0067	− 0.0028	0.0007
0.2003	0.879	0.886	0.892	0.898	− 0.0110	− 0.0065	− 0.0025	0.0008
0.2503	0.880	0.887	0.892	0.898	− 0.0110	− 0.0061	− 0.0023	0.0014
0.2993	0.880	0.887	0.892	0.899	− 0.0110	− 0.0058	− 0.0020	0.0019
*m*_*DL-Alanine*_ = 0.1499 mol kg^−1^								
0.0506	0.883	0.889	0.895	0.901	− 0.0110	− 0.0070	− 0.0030	− 0.0002
0.1007	0.882	0.889	0.894	0.901	− 0.0110	− 0.0060	− 0.0021	0.0008
0.1520	0.881	0.889	0.893	0.9	− 0.0110	− 0.0059	− 0.0022	0.0010
0.2023	0.881	0.888	0.893	0.899	− 0.0110	− 0.0057	− 0.0019	0.0010
0.2513	0.88	0.888	0.893	0.898	− 0.0100	− 0.0055	− 0.0019	0.0012
0.3030	0.88	0.887	0.892	0.897	− 0.0100	− 0.0054	− 0.0017	0.0019
*m*_*L-Proline*_ = 0.0501 mol kg^−1^								
0.0481	0.877	0.884	0.891	0.898	− 0.0120	− 0.0076	− 0.0040	0.0021
0.0981	0.879	0.885	0.895	0.901	− 0.0110	− 0.0068	− 0.0026	0.0036
0.1476	0.879	0.886	0.895	0.904	− 0.0110	− 0.0066	− 0.0033	0.0031
0.1950	0.879	0.886	0.895	0.903	− 0.0110	− 0.0065	− 0.0026	0.0037
0.2442	0.879	0.886	0.894	0.900	− 0.0110	− 0.0064	− 0.0022	0.0037
0.2912	0.88	0.887	0.894	0.901	− 0.0110	− 0.0066	− 0.0024	0.0031
*m*_*L-Proline*_ = 0.0962 mol kg^−1^								
0.0498	0.878	0.884	0.889	0.897	− 0.0130	− 0.0079	− 0.0044	− 0.0008
0.0997	0.876	0.886	0.89	0.899	− 0.0122	− 0.0062	− 0.0026	0.0007
0.1502	0.879	0.887	0.892	0.899	− 0.0113	− 0.0056	− 0.0025	0.0004
0.1992	0.879	0.886	0.892	0.900	− 0.0109	− 0.0056	− 0.0025	0.0014
0.2495	0.880	0.886	0.893	0.900	− 0.0105	− 0.0054	− 0.0020	0.0007
0.3112	0.884	0.891	0.896	0.902	− 0.0084	− 0.0034	− 0.0004	0.0023
*m*_*L-Proline*_ = 0.1488 mol kg^−1^								
0.0486	0.879	0.885	0.891	0.896	− 0.0104	− 0.0060	− 0.0021	0.0005
0.0991	0.879	0.886	0.891	0.896	− 0.0101	− 0.0047	− 0.0009	0.0016
0.1477	0.879	0.886	0.891	0.895	− 0.0099	− 0.0050	− 0.0012	0.0010
0.1997	0.879	0.886	0.891	0.895	− 0.0101	− 0.0051	− 0.0015	0.0012
0.2492	0.880	0.886	0.892	0.896	− 0.0099	− 0.0049	− 0.0013	0.0011
0.2955	0.879	0.886	0.891	0.896	− 0.0099	− 0.0049	− 0.0013	0.0013

The standard uncertainties for molality, temperature and pressure were* u* (*m*) = 0.002 mol kg^−1^, *u* (*T*) = 0.02 K, *u* (*P*) = 10 hPa, respectively with level of confidence 0.68.

The *ASV* value have been introduced as sweetness criterion in the literature. Where, acceptable values for this property should be 0.5 < *ASV* < 0.7 is an ideal range of sweetness while the reported data show higher value rather than this value. Also, the *ASIC* has been studied that is in agreement with the observed trend as *ASV*. However, it should be diluted to the lower concentration of the solution to achieve an appropriate grade. Previous studies have been deducted that using choline chloride would be increase salt intake and would enhance taste of the salt^41^. However, the amino acid supplements are usually used for animal food and it has considerable salt ingredient in the mixture. Accordingly, it could be enhancing the amino acid supplementation in the supply chain and improve the quality of the food.

Previous studies have indicated that using choline chloride can increase salt intake and enhance the taste of salt. This is a significant observation, as choline chloride is commonly used as a supplement. In the context of amino acid supplements, which are typically used for animal food, the mixture usually contains a considerable amount of salt. The presence of choline chloride in these supplements could therefore enhance the overall taste due to its salt-enhancing properties. The implications of these findings are noteworthy for the supply chain of amino acid supplements. By incorporating choline chloride, the supplementation process can be enhanced, potentially improving the quality of the food provided to animals. This enhancement comes from the improved taste, which could make the food more palatable and thus more likely to be consumed in adequate amounts by the animals. In summary, the *ASV* values for the studied solutions are higher than the ideal range, suggesting an overly sweet taste. The *ASIC* values support this trend, indicating an increased saltiness. Diluting the solutions to lower concentrations is recommended to achieve the appropriate sweetness and saltiness levels. The use of choline chloride not only increases salt intake but also enhances the taste of salt, which can be beneficial in amino acid supplements used in animal food. This can improve the quality and acceptability of the food, ultimately enhancing the supplementation process in the supply chain.

### Hydration behavior with COSMO and ePC-SAFT

The σ-profile is essential in COSMO-based thermodynamics, reflecting the charge distribution on a molecule's surface. Acting as a unique fingerprint, it shows the likelihood of specific charge density values in segmented segments. COSMO models, like COSMO-RS and COSMO-SAC, use σ-profiles to predict thermodynamic properties and molecule-environment interactions. These profiles are typically obtained via density functional theory (DFT) calculations, which can be computationally intensive. To mitigate this, alternative methods for approximating σ-profiles are available in software tools and databases, facilitating quicker analysis, especially useful in high-throughput screening applications.

**Figure 2:**
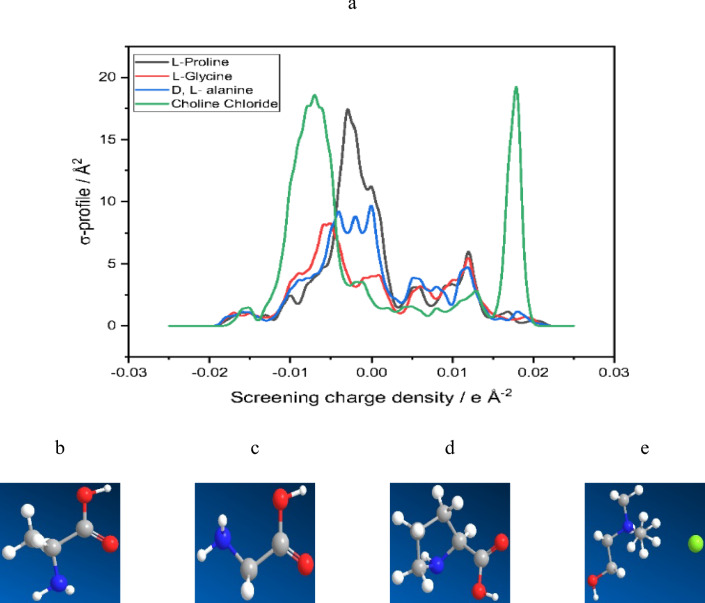
(**a**) The σ-profiles of amino acids, choline chloride, and optimized structures of (**b**) l-glycine, (**c**) d,l-alanine, (**d**) l-proline, (**e**) choline chloride.

The GGA VWN-BP function in Dmol3 is recommended by the developer and has shown promising results for real solvents. In this study, COSMO results were obtained through DFT calculations using the Dmol3 module of Materials Studio (Biovia, Materials Studio 2023). Molecule geometries were optimized using GGA (VWN-BP). Figure 2 shows the optimized structures and corresponding COSMO results, specifically the σ-profiles, for the solvents and IL under investigation. Table 5 provides the cavity volume, cavity surface area, and the HOMO and LUMO energies of the compounds from Dmol3 energy optimization calculations. The GGA VWN-BP function, a crucial exchange correlation functional used in DFT calculations. It is employed in the Dmol3 module of Materials Studio for electronic structure calculations and simulations. This function combines the exchange functional of Vosko, Wilk, and Nusair with the correlation functional developed by Becke and Perdew. The exchange part facilitates electron exchange between orbitals, while the correlation part accounts for electron interactions. By integrating these components, the VWN-BP function approximates the exchange correlation effects in the system. As a GGA functional, it considers not only the electron density but also its gradient, providing a more accurate representation of the system's electronic structure compared to simpler functionals like the local density approximation (LDA). In Dmol3, the GGA VWN-BP function optimizes molecular geometry, computes electronic properties, and performs various DFT calculations, aiming for reliable and precise outcomes in diverse applications, including solvation behavior in real solvents. In this study, water was chosen as the solvent.

**Table 5:** The results of COSMO calculations including surface area and total volume of cavity, besides the total HOMO and LUMO orbitals number and energy and dielectric (solvation energy) in water.

Compound	Surface area of cavity, Å^2^	Total volume of cavity, Å^3^	Dielectric (solvation) energy, kcal/mol	n_HOMO_	n_LUMO_	E_HOMO_, eV	E_LUMO_, eV
l-glycine	106.9712	86.7222	− 13.06	20	21	− 5.70	− 1.07
d,l-alanine	123.0834	105.1876	− 12.76	24	25	− 5.72	− 1.13
l-proline	144.7045	132.6716	− 11.98	31	32	− 5.41	− 1.08
Choline chloride	186.1378	174.8311	− 56.03	38	39	− 5.07	0.40

l-Proline has the smallest cavity volume and surface area among the molecules, indicating a more compact solute. It also has the least negative dielectric (hydration) energy, suggesting weaker interactions with the solvent compared to other molecules. The HOMO energies for all molecules are negative, while the LUMO energies vary across the compounds.

The modeling of the density of the aqueous solutions of choline chloride and amino acids have been carried out with ePC-SAFT equation of state. The general form of the equation of state could be represented with free Helmholtz energy term by the following relation,

21 $$a = a^{hc} + a^{disp} + a^{assoc} + a^{ion}$$

where the superscripts *hc* and *disp* show the hard chain and dispersion force. The symbol *assoc* was used for association contribution that uses the original SAFT equations. In the case of studied systems, the *2B* approach for amino acids and 4C for H_2_O were used. The *dipole* is represented for the polar contribution (dipole momentum) of the components. The *ion* term has been calculated based on the PC-SAFT electrolyte extension term. The density could be predicted using the following equation with iteration of pressure, The utilized parameters segment number, *m*, segment diameter, *σ*, dispersion energy, *ε/K*_*B*_, association energy, *ε*_*AiBi*_*/K*_*B*_ and effective association volume, *κ*_*AiBi*_ are given in Table 6.

**Table 6:** The comparison of ePC-SAFT parameters of the studied compounds calculated from the experimental data (Exp.) and COSMO calculations.

Compound	Evaluation method	*m*	*σ*	*ε/K*_*B*_,	*κ*_*AiBi*_	*ε*_*AiBi*_*/K*_*B*_	*Ref*
Choline	Exp.	1	5.9216	220.4883	–	–	^30^
Chloride	Exp.	1	3.0575	47.2878	–	–	^30^
ChCl	COSMO	7.462	2.818	2796	0.02	18,190	
l-Glycine	Exp.	4.8495	2.3270	216.96	0.0393	2598.1	^32^
	COSMO	5.756	2.432	7324	0.02	41,260	
d,l-Alanine	Exp.	5.4647	2.5222	287.59	0.0819	3176.6	^42^
	COSMO	5.96	2.564	5230	0.02	30,590	
l-Proline	Exp.	6.9811	2.5481	289.72	0.0362	5527.8	^42^
	COSMO	6.088	2.751	3294	0.0	20,730	
Water	Exp.	1.204659	*a*	353.9449	0.04509	2425.7	^30^

$$a = 2.7927 + 10.11\exp \left( { - 0.01775T} \right) - 1.417\exp \left( { - 0.01146T} \right)$$.

The given parameters in Table 6 have been used to predict the density of the aqueous solutions of choline chloride in the presence of different concentrations of studied amino acids at different temperatures. The corresponding average relative deviations of these systems are given in Table 7. As could be seen the COSMO-based parameters are not suitable for predictive calculation of the density of the studied systems. However, according to the results the ePC-SAFT model is a successful model in the prediction of density of the studied solutions while the alanine solutions show poor results in the prediction, the proline solutions predicted with high accuracy with experimental data. Accordingly, this equation of state could be used to evaluate the energy of the solvation of these solutions. Also, this equation of state could provide other calorimetric properties of the systems such as enthalpy. These results could be used to interpret the interactions between the existing species in the solutions.

**Table 7:** The average relative deviation percent of the predicted densities of the aqueous solutions containing choline chloride in the presence of different concentrations of the studied amino acids (l-glycine, d,l-alanine and l-proline) at different temperatures.

T/K	ARD %					
	Exp	COSMO	Exp.	COSMO	Exp.	COSMO
	0.05 m _L_-Glycine in water + ChCl			0.10 m _L_-Glycine in water + ChCl		0.15 m _L_-Glycine in water + ChCl
288.15	3.54	11.54	3.52	11.60	3.51	11.74
298.15	3.69	11.67	3.9	11.98	3.89	12.12
308.15	3.73	11.73	4.27	12.35	4.26	12.49
318.15	3.69	11.67	4.64	12.72	4.64	12.87
	0.05 m _D,L_-Alanine in water + ChCl			0.10 m _D,L_-Alanine in water + ChCl		0.15 m _D,L_-Alanine in water + ChCl
288.15	8.52	16.51	8.47	16.55	8.43	16.66
298.15	8.38	16.37	8.34	16.42	8.30	16.53
308.15	7.75	15.74	8.3	16.38	8.27	16.50
318.15	8.41	16.40	8.36	16.44	9.45	17.68
	0.05 m _L_-Proline in water + ChCl			0.10 m _L_-Proline in water + ChCl		0.15 m _L_-Proline in water + ChCl
288.15	0.31	8.31	0.33	8.41	0.28	8.51
298.15	0.12	8.12	0.16	8.24	0.13	8.36
308.15	0.46	8.46	0.56	8.64	0.47	8.70
318.15	0.72	8.72	0.87	8.95	0.73	8.96

The study compares two models: COSMO-based parameters and the ePC-SAFT model. The COSMO-based parameters are derived from the Conductor-like Screening Model approach, while the ePC-SAFT model is an equation of state tailored for predictive calculations. The amino acids studied include l-glycine (Gly), d,l-alanine (Ala), and l-proline (Pro). The temperatures examined range from 288.15 to 318.15 K, and the concentrations of amino acids in the water + ChCl solutions are 0.05 m, 0.10 m, and 0.15 m. The average relative deviation percent (ARD %) values represent the average relative deviation of the predicted densities from the experimental values, with lower ARD % values indicating better predictive accuracy. For l-glycine solutions, the ePC-SAFT model shows ARD % values around 3.5% to 4.64%, indicating relatively good predictive accuracy. In contrast, the COSMO-based parameters show significantly higher ARD % values (around 11.54% to 12.87%), suggesting poor predictive performance for these systems. For d,l-alanine solutions, the ePC-SAFT model shows ARD % values around 7.75% to 9.45%. These values are higher compared to those for _L_-glycine solutions, indicating poorer predictive accuracy for Ala solutions. The COSMO-based parameters again show higher ARD % values (around 15.74% to 17.68%), indicating poor predictive performance. The ePC-SAFT model shows exceptionally low ARD % values (ranging from 0.12 to 0.87%) for l-proline solutions, indicating excellent predictive accuracy. The COSMO-based parameters show ARD % values around 8.12% to 8.96%, which are significantly higher than those for the ePC-SAFT model, indicating poor predictive performance. In summary, the COSMO-based parameters are generally not suitable for predicting the density of the studied systems as they consistently show high ARD % values across all amino acids and temperatures. The ePC-SAFT model proves to be successful in predicting the density of the studied ternary solutions, particularly for l-proline-based solutions, which show very low ARD % values indicating high accuracy. For l-glycine-based solutions, the model also performs reasonably well, but it shows poorer results for d,l-alanine-based solutions. These results imply that the ePC-SAFT model can be used confidently for predicting the density of ternary solutions, especially those containing l-proline. This model may also be useful for calculating other thermodynamic properties such as enthalpy and solvation energy. The good performance of the ePC-SAFT model for l-proline solutions suggests that the model accurately captures the interactions between choline chloride, water, and l-proline. The poorer performance for d,l-alanine may indicate more complex interactions that are not as well captured by the ePC-SAFT model. For systems where the ePC-SAFT model does not perform well (e.g*., *d,l-alanine), further refinement or alternative models might be necessary to achieve better predictive accuracy.

## Conclusion

l-glycine, d,l-alanine, l-proline, choline chloride, and water physicochemical properties of ternary solutions were studied. The results indicate that increasing the concentration of amino acids and choline chloride increase solution density, wherea at higher temperatures reduce it. The apparent molar volume (*Vφ*) and apparent molar isentropic compressibility (*κφ*) values indicates underscore significant solute–solvent and solute–solute interactions, with *Vφ* increase and *κφ* falling in response to increased solute concentration and temperature, indicative of increased solution and compression. Thermodynamic results are obtained from the Redlich-Mayer model confirmed that choline chloride acts as a structure breaker in these solutions, as evidenced by the apparent molar thermal expansion (*α*) and Hepler's structural parameter. The apparent specific volume and apparent specific isentropic compressibility suggested that although the ASV values were outside the ideal sweetness range, dilution could potentially improve the taste profile by reducing the salinity imparted by the solutes. Advanced computational methods, including COSMO-based thermodynamic and ePC-SAFT (electrolyte Perturbed-Chain Statistical Associating Fluid Theory), provided robust predictions of molecular interactions and solution properties. While the ePC-SAFT model demonstrated high accuracy for the L-proline solutions, it was less accurate for the _D,L-_alanine solutions. Nevertheless, it proved effective in predicting densities and evaluating solvation energies and other calorimetric properties, offering valuable insights into the molecular dynamics of these complex solutions. Overall, this study enhances our understanding of the intricate interactions within ternary solutions of amino acids, choline chloride, and water, with implications for tailoring solution properties in various applications, including food science and biotechnology.

### Supplementary Information

Supplementary Information.

## Data Availability

All data generated or analysed during this study are included in this published article [and its supplementary information files].
