# Supplementary material for: Choline chloride and amino acid solutions taste and hydration behavior with experimental thermodynamic properties and COSMO-PC-SAFT calculation
Source: Sci Rep. 2024 Sep 2;14:20372. doi: 10.1038/s41598-024-70275-z (PMC11368939; doi:10.1038/s41598-024-70275-z)
Supplement: Supplementary file 1 — Supplementary Information. [file 41598_2024_70275_MOESM1_ESM.docx]

Supporting Information

**Choline Chloride and Amino Acid Solutions Taste and Hydration Behavior with Experimental Thermodynamic Properties and COSMO-PC-SAFT Calculation**

Mohammad Amin Morsali, Behrang Golmohammadi, and Hemayat Shekaari*

*Department of Physical Chemistry, Faculty of Chemistry, University of Tabriz, 29 Bahman Boulevard, Tabriz, Iran, Tel.: +98-41-33393139, E-mail:* *hemayatt@yahoo.com*

**Figure S1.** The a) density and b) speed of sound of aqueous solutions of *L*-Glycine at different temperature compared with literature data. ^1^

**Figure S2.** The a) density and b) speed of sound of aqueous solutions of *D, L*-Alanine at different temperature compared with literature data. ^1^


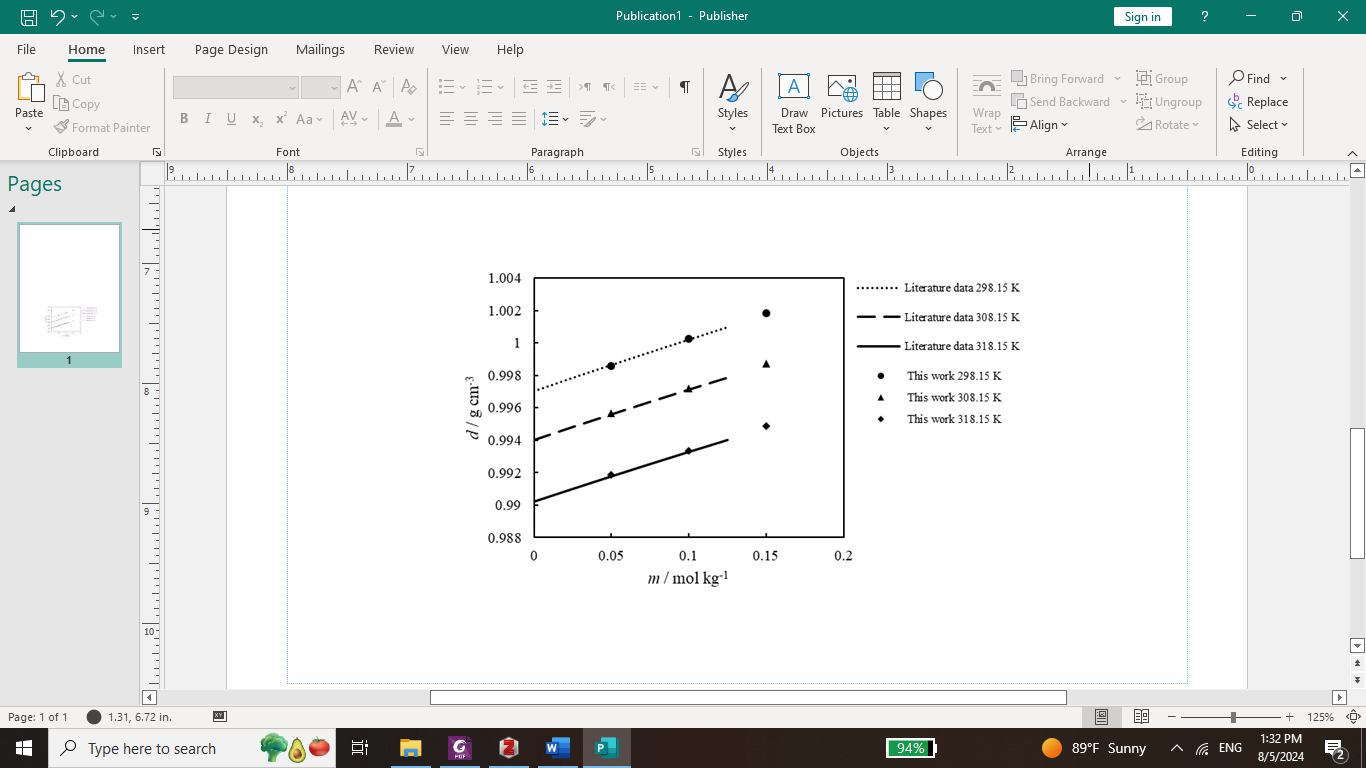


**Figure S3.** The density of aqueous solutions of *L*-Proline at different temperature compared with literature data.^2^

**References**

1. Kumar, H., Singla, M. & Jindal, R. Interactions of glycine, L-alanine and L-valine with aqueous solutions of trisodium citrate at different temperatures: A volumetric and acoustic approach. *The Journal of Chemical Thermodynamics* **67**, 170–180 (2013).

2. Nain, A. K. Insight into solute-solute and solute-solvent interactions of *l*-proline in aqueous-D-xylose/L-arabinose solutions by using physicochemical methods at temperatures from 293.15 to 318.15 K. *Journal of Molecular Liquids* **318**, 114190 (2020).
